# Enhanced Hydrogen Evolution in the Presence of Plasmonic Au-Photo-Sensitized g-C_3_N_4_ with an Extended Absorption Spectrum from 460 to 640 nm

**DOI:** 10.1371/journal.pone.0161397

**Published:** 2016-08-30

**Authors:** Lihong Xie, Zhuyu Ai, Meng Zhang, Runze Sun, Weirong Zhao

**Affiliations:** Department of Environmental Engineering, Zhejiang University, Hangzhou, 310058, China; Institute of Materials Science, GERMANY

## Abstract

Extensively spectral-responsive photocatalytic hydrogen production was achieved over g-C_3_N_4_ photo-sensitized by Au nanoparticles. The photo-sensitization, which was achieved by a facile photo-assisted reduction route, resulted in an extended spectral range of absorption from 460 to 640 nm. The photo-sensitized g-C_3_N_4_ (Au/g-C_3_N_4_) photocatalysts exhibit significantly enhanced photocatalytic hydrogen evolution with a TOF value of 223 μmol g^-1^ h^-1^, which is a 130-fold improvement over g-C_3_N_4_. The hydrogen production result confirms that Au nanoparticles are effective photo-sensitizers for the visible light-responsive substrate g-C_3_N_4_. UV–vis diffuse reflection spectra (DRS), photoluminescence spectra (PL), electron spin resonance (ESR), and electrochemical measurements were used to investigate the transfer process of photogenerated electrons. The optimal Au/g-C_3_N_4_ photocatalyst displays the lowest charge transfer resistance of 18.45 Ω cm^-2^ and a high electron transfer efficiency, as determined by electrochemical impedance spectroscopy (EIS). The photo-sensitized g-C_3_N_4_ shows a broad range of response to visible light (400–640 nm), with significantly high incident photon-to-current efficiency (IPCE) values of 14.52%, 2.9%, and 0.74% under monochromatic light irradiation of 400, 550, and 640 nm, respectively. ESR characterization suggests that Au nanoparticles are able to absorb visible light of wavelengths higher than 460 nm and to generate hot electrons due to the SPR effect.

## Introduction

In the past few decades, the development of photoinduced procedures for hydrogen production has been a cutting-edge research area due to the growing expectation for clean energy production and the increasingly serious energy crisis. [1–4] Photocatalytic production of hydrogen, employing semiconductor catalysts may be the most promising albeit challenging approach because of its potential application in the direct production of clean energy by utilizing water and inexhaustible solar energy. Various semiconductor photocatalysts, such as TiO_2_, ZnO, and SrTiO_3_ have been widely studied for the photocatalytic production of hydrogen from water splitting. [5–9] These semiconductor photocatalysts have been so far demonstrated to be active in water splitting, however most of them are responsive to UV irradiation only. The relatively low quantum efficiency and extremely insufficient utilization of solar light restrict further application of semiconductor catalysts. [10] To address these limitations, much effort has been focused on the development of second-generation catalysts with a broader response to visible light.

Recently, a novel polymer n-type semiconductor, layered C_3_N_4_ with a graphitic structure (g-C_3_N_4_) has attracted much attention, showing suitable energy band gap (*E*_g_ = 2.7 eV) and photocatalytic stability for hydrogen production. Compared with the most studied materials, g-C_3_N_4_ combines the advantages of low cost, nontoxicity and visible light activity, and in this regard, it should be a good candidate for photocatalytic solar conversion. However, pure g-C_3_N_4_ can only absorb blue light up to 460 nm, [11] which limits the utilization of solar energy. A variety of approaches have been used to modify g-C_3_N_4_ to extend the range of visible light absorption, such as doping g-C_3_N_4_ with other elements, [12] sensitization by quantum dots, [13] and coupling with other semiconductor photocatalysts. [14] Ge et al. [12] introduced impurities into g-C_3_N_4_ by doping it with S and found that the region of visible light absorption was extended from 460 to 500 nm. Dong et al. [15] actualized carbon self-doping of g-C_3_N_4_ via calcinating of solvothermally treated melamine with absolute alcohol, which increased the visible-light absorption and decreased band gap of g-C_3_N_4_ from 2.72 to 2.65 eV. Ge et al. [13] reported that sensitizing g-C_3_N_4_ with CdS quantum dots greatly improved its photocatalytic activity for hydrogen evolution and caused a red shift; the visible light absorption region was shifted to 580 nm. Despite these improvements, S-doped g-C_3_N_4_, C-doped, as well as g-C_3_N_4_ sensitized with CdS quantum dots showed an absorption edge at 500 or 580 nm, which is mismatched with the visible light region of approximately 580–640 nm. In addition, doping introduces trap states and induces charge carrier recombination, and CdS is a toxic material which is easily photo-corroded. [16] Therefore, it is urgent to develop materials that are secure and efficient and that show excellent response to visible light; these properties are desirable to extend the absorption spectrum to longer wavelengths, so that such materials can be utilized in the photocatalytic evolution of hydrogen from water.

The modification of semiconductor nanoparticles by noble metal catalysts has been extensively studied because it is one of the most promising methods to broaden the visible-light response. [17, 18] Noble metals such as Au and Ag, which possess excellent absorption in the visible light region, have been used as photocatalysts due to the surface plasmon resonance (SPR) effect. [19–21] SPR refers to the collective oscillation of surface electrons, with an oscillation frequency that is dependent on the size, shape, and nanostructure of the metal. [22, 23] Pawar et al. [24] reported that Au nanoparticles substantially increase the light absorption of g-C_3_N_4_ from 460 to 700 nm, leading to enhanced photocatalytic activity. Chang et al. [25] verified that nanoparticles of the noble metal Pd deposited on the surface of g-C_3_N_4_ facilitated the separation of photoinduced charge carriers and showed strong absorption in the visible light region from 400 to 700 nm. Silva et al. [26] found that the use of Au nanoparticles as photo-sensitizers on the surface of s increased hydrogen evolution under irradiation with monochromatic visible light (*λ* = 532 nm) in the presence of such Au/TiO_2_ catalysts. This wavelength is above the TiO_2_ absorption edge and coincides approximately with the maximum of the Au-SPR absorption band. Thus, it is reasonable to assume that Au nanoparticles can be used to harvest visible light due to their SPR. On the other hand, the relatively low Fermi level of Au nanoparticles leads to electron transfer from the semiconductor conduction band (CB) to the Au nanoparticles, which should suppress the recombination of charge carriers. Considering the advantages of Au nanoparticles, it is expected that the photocatalytic performances of semiconductor photocatalysts can be further improved by loading them with Au nanoparticles.

Based on the above analysis, it is proposed that the modification of g-C_3_N_4_ by Au nanoparticles can improve the electron–hole separation of g-C_3_N_4_ and extend the absorption region to the visible light region. At the same time, g-C_3_N_4_, acting as a substrate can enhance the dispersion of Au nanoparticles. [27] The hybrid plasmonic structure developed here exhibits significantly improved visible-light-driven activity for photocatalytic hydrogen evolution under visible light illumination (400 < *λ* < 640 nm). The enhancement of hydrogen production in the presence of Au/g-C_3_N_4_ is discussed in this paper. The SPR effect of Au nanoparticles, lower electron transfer resistance, high IPCE value, broader visible light response, and higher intensity of trapped electrons, all lead to a superior photocatalytic activity; the mechanism of which is discussed in this work.

## Materials and Methods

### Materials

Dicyandiamide (C2H4N4), acetone (CH3COCH2COCH3), chloroauric acid tetrahydrate (AuCl3·HCl·4H2O), methanol (CH3OH), ethanol (C2H5OH), and sodium sulfate anhydrous (Na2SO4) were purchased from Sinopharm Chemical Reagent Co., Ltd, China. All reagents were analytical grade and used without further purification. Deionized water was used for all experiments.

### Preparation of photocatalysts

The fabrication procedure of g-C_3_N_4_ was based on previous work. [28] Typically, 10 g of dicyandiamide, placed in a crucible with a cover under ambient pressure at room temperature, was heated at a rate of 10°C min^-1^ to reach a temperature of 500°C and then calcined at this temperature for 2 h. The product was heated further at a rate of 5°C min^-1^ to reach a temperature of 520°C and then calcined at this temperature for another 2 h. After naturally cooling to room temperature, g-C_3_N_4_ was ground into a powder and collected through a 200 mesh sieve. About 6 g of photocatalysts was made.

The photocatalyst Au/g-C_3_N_4_ with different amounts of Au nanoparticles was synthesized by a facile, photo-assisted reduction method. The typical procedure was as follows: g-C_3_N_4_ (0.30 g) was dispersed in a methanol/water (1:4 v/v) solution with a total volume of 75 mL in a double-layer photoreaction cell. A certain volume of AuCl_3_·HCl·4H_2_O solution (10 mg mL^-1^) was then added to the cell as the gold precursor, and magnetic stirred for 2 h to mix homogeneously. The resulting suspension was irradiated under a 300 W Xe lamp with a wavelength range of 200–400 nm for 3 h under continuous stirring. The final product was separated by filtration, washed three times with distilled water, and dried in an oven at 60°C for 12 h. Following this method, Au/g-C_3_N_4_ samples with different weight ratios of 0.2%, 0.5%, 1.0%, 2.0%, and 5.0% were obtained and designated as S_0.2_, S_0.5_, S_1_, S_2_, and S_5_, respectively. The g-C_3_N_4_ sample was designated as S_0_.

### Materials characterization

The crystal structure of the photocatalysts was analyzed by X-ray diffraction (XRD, XRD-6000, Shimadzu, Japan) with Cu K*α* radiation (*λ* = 0.1546 nm), at a scan rate of 10° min^-1^. The accelerating voltage and the applied current were 40 kV and 40 mA, respectively.

Transmission electron microscopy (TEM, TecnaiG2 F20S-TWIN, USA) and high-resolution transmission electron microscopy (HRTEM) at an accelerating voltage of 200 kV were used to characterize the morphology and structure of the obtained products. The pretreatment of samples were as follows: A certain amount sample was dispersed in an absolute ethyl alcohol solution followed by sonication for 20 min. After dispersed uniformly, the sample was drawn by capillary tube to a cupper grid and air dried.

X-ray photoelectron spectroscopy (XPS) was performed on a Thermo Escalab 250 instrument with a monochromatic Al K*α* source (1486.71 eV). The binding energy scale was calibrated with respect to the C1s peak at 284.6 eV.

The surface areas of samples were determined by the Brunauer Emmett Teller (BET) method using N_2_ adsorption–desorption isotherms at 77 K by a surface area analyzer (3H-2000 PSII, Beishide Intrument, China). The samples were degassed 3 h before BET measurements.

UV–vis diffuse reflection spectra (DRS) of the dry-pressed disk samples were obtained with a UV–vis spectrometer (TU-1901, Pgeneral, China), using BaSO_4_ as a reference. The spectra were recorded in the wavelength range from 230 to 850 nm.

Photoluminescence (PL) was measured at room temperature on a fluorescence spectrophotometer (Fluorolog-3-Tau, France) with an excitation wavelength of 325 nm. The widths of the excitation and emission slits were 5.0 nm.

Electron spin resonance (ESR) spectra were recorded using a Jeol JES FA200 spectrometer at a temperature of 90 K.

Quantitative elemental analyses of Au were carried out in an inductively coupled plasma-atomic emission spectrometer (ICP-AES, Prodigy, Leeman, USA).

### Electrochemical measurements

The electrochemical properties of all samples were measured using an electrochemical workstation (CH Instruments 650D, China) in a standard three-electrode setup with Pt mesh as the counter electrode and Ag/AgCl as the reference electrode. The electrolyte was 120 mL of 0.1 mol L^-1^ Na_2_SO_4_ aqueous solution. Before measurement, the electrolyte was purged with pure N_2_ for 30 min to remove dissolved oxygen. A high-pressure Xe lamp (300 W) equipped with a VisREF (350–780 nm) and an UVIRCUT (400–780 nm) filter was used as the light source, which provided visible light in the wavelength range of 400–780 nm. The working electrode was constructed by screen-printing indium–tin oxide (ITO) glass with g-C_3_N_4_ and Au/g-C_3_N_4_ samples. The preparation procedure was as following: 1 g of photocatalysts was dissolved in the mixture of 1 mL deionized water and 0.05 mL acetyl acetone. Then the slurry was screen-printing in indium–tin oxide (ITO) glass electrode and the area of the photocatalyst printed was kept on 1 cm^2^. Finally, the electrode was calcined at 300°C for 1 h as the working electrode.

The electrochemical impedance spectroscopy (EIS) data was obtained in the frequency range from 100000 to 100 Hz at an amplitude of 10 mV in the dark, under open-circuit conditions. Amperometric i-t curves were obtained using the same electrochemical device by alternately turning the light on and off. The incident photon-to-current efficiency (IPCE), used to investigate the photoresponsivity, was measured using the Xe lamp with specific wavelength filters to select the required wavelength of light.

### Photocatalytic hydrogen production

The g-C_3_N_4_ and Au/g-C_3_N_4_ samples were tested for the production of hydrogen from water under visible light irradiation (*λ* ≥ 400 nm). Photocatalytic hydrogen production was carried out in in a top-illuminated, jacketed quartz photoreactor. In the typical experiment, 0.05 g of photocatalysts was dispersed in methanol/water (3:7 v:v) solution as a sacrificial agent for hydrogen production. The concentration of photocatalysts was 1 g L^-1^. The solution was continuously stirred with a magnetic stirrer. Before measurement, argon was purged through the suspension for 30 min to remove oxygen. The reaction system was irradiated using a 300 W Xe lamp equipped with a VisREF (350–780 nm) and an UVIRCUT (400–780 nm) filter. The amount of hydrogen produced was determined by gas chromatography (Fuli 9790, China), using a thermal conductivity detector (TCD).

## Results and Discussion

### X-ray diffraction

**Fig 1:**
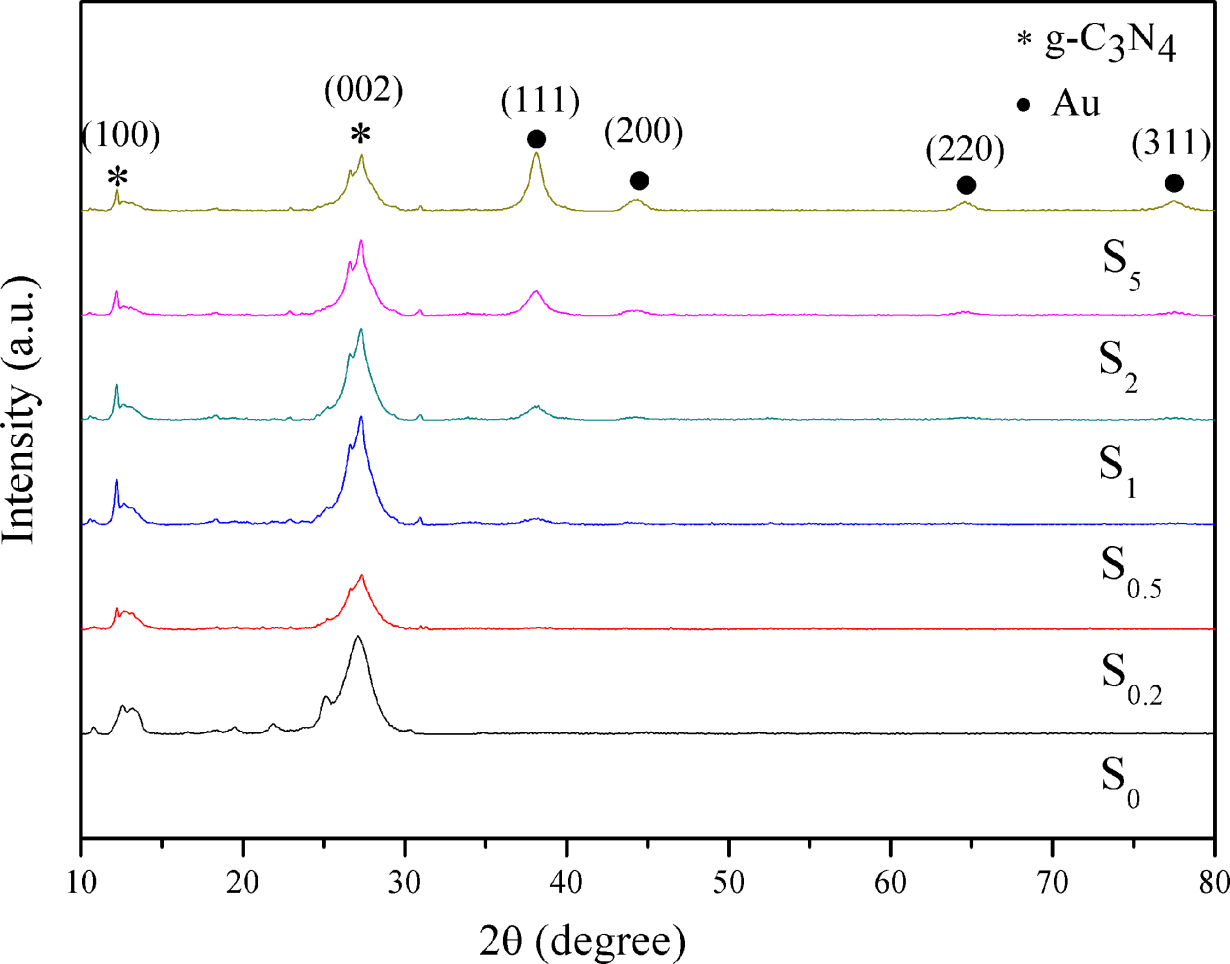
XRD patterns of S_0_, S_0.2_, S_0.5_, S_1_, S_2_, and S_5_ samples.

The crystal structures and the possible phase changes of the samples are examined by XRD. Fig 1 shows the XRD patterns of S_0_, S_0.2_, S_0.5_, S_1_, S_2_, and S_5_ samples. All the samples present similar profiles. S_0_ displays two pronounced diffraction peaks corresponding to the (100) and (002) planes of g-C_3_N_4_, at 2θ of approximately 13.04° and 27.34°, respectively. The diffraction peak at 2θ of approximately 13.04° can be ascribed to the characteristic inter-layer structural packing. And the diffraction peak at 2θ of 27.34° is attributed to the interlinear stacking peaks of the aromatic systems, respectively. [29, 30] This confirms that g-C_3_N_4_ was successfully synthesized by thermal depolymerization method. The two diffraction peaks can also be clearly observed in the Au/g-C_3_N_4_ samples with different Au loading amounts. After the deposition of Au nanoparticles, new diffraction peaks are observed at 38.16°, 44.54°, 64.84°, and 77.52°. These are, respectively, the typical peaks for the (111), (200), (220), and (311) planes of Au (JCPDS: 04–0784) nanoparticles, indicating their deposition on the g-C_3_N_4_ surface. The intensity of the (111) peak is much stronger than those of other peaks, suggesting that Au (111) plane is the predominant crystal facet in the synthesized Au nanoparticles. In the S_0.2_, S_0.5_, S_1_, S_2_, and S_5_ samples, the characteristic peaks of Au become stronger as the Au-content is increased. When the content of Au nanoparticles is 0.2 wt % and 0.5 wt %, the diffraction peaks related to Au are negligible. This can be attributed to the low weight-loading of Au nanoparticles on the surface of g-C_3_N_4_.

### Transmission electronic microscopy

**Fig 2:**
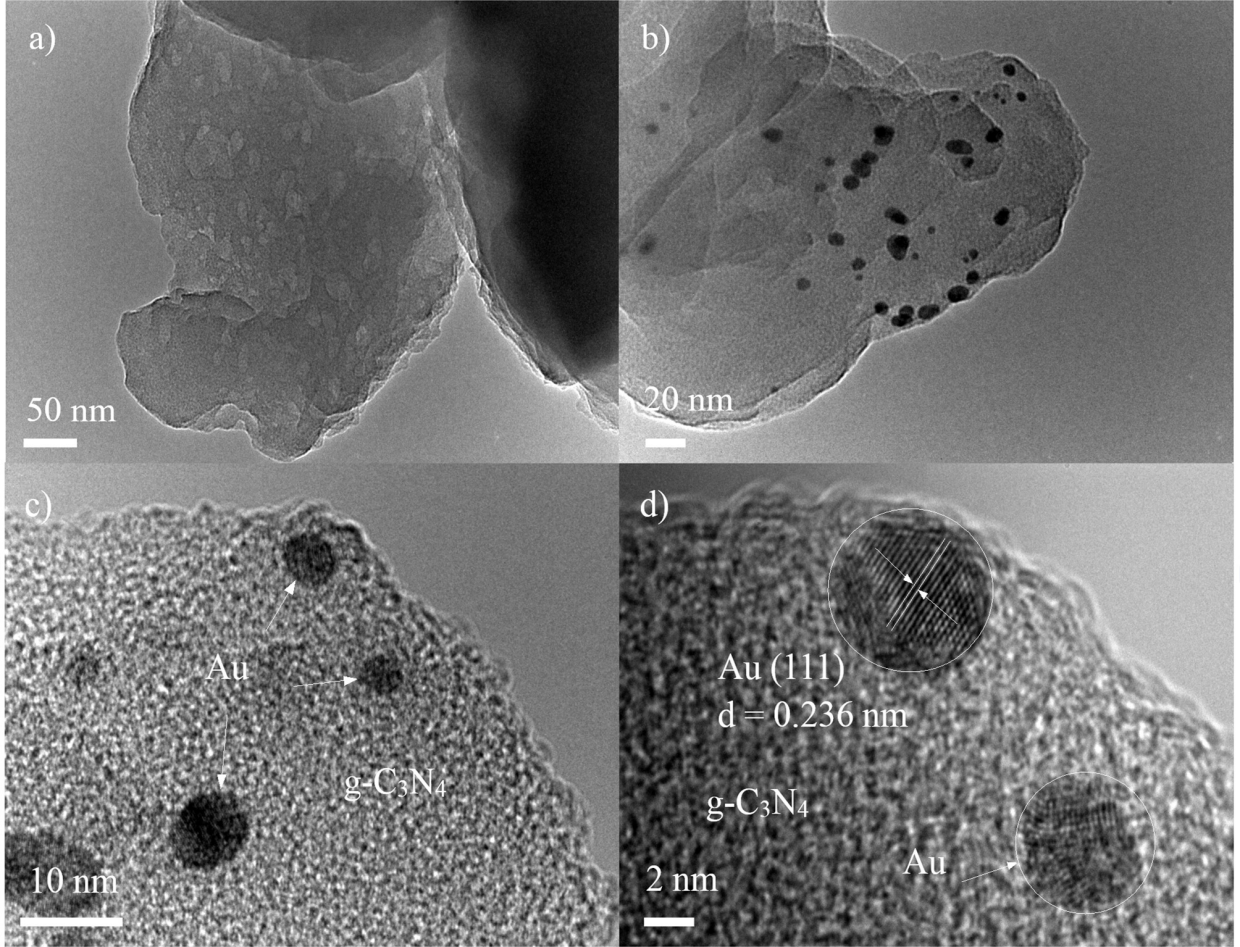
TEM images of S_0_ (a), S_1_ (b, c), and HRTEM of the S_1_ sample.

TEM and HRTEM are used to investigate the morphology and microstructure of the samples. As shown in Fig 2A, a typical aggregated morphology with a large size and lamellar structure can be observed for S_0_. Moreover, there are many mesopores existing in those lamellar structures due to the different size of each layer of g-C_3_N_4_. Fig 2B and 2C show a typical TEM image of the S_1_ sample, which presents the best photocatalytic performance for hydrogen production. It is evident that some of black-colored dots with an average size of approximately 5 nm, corresponding to Au nanoparticles, are distributed on the g-C_3_N_4_ surface. The Au nanoparticles contact with g-C_3_N_4_ closely, which suggests Au nanoparticles are successfully loaded on the surface of g-C_3_N_4_. Fig 2D is a HRTEM image of the S_1_ sample. The lattice spacing is 0.236 nm, which corresponds to the Au (111) plane.

### X-ray photoelectron spectroscopy

**Fig 3:**
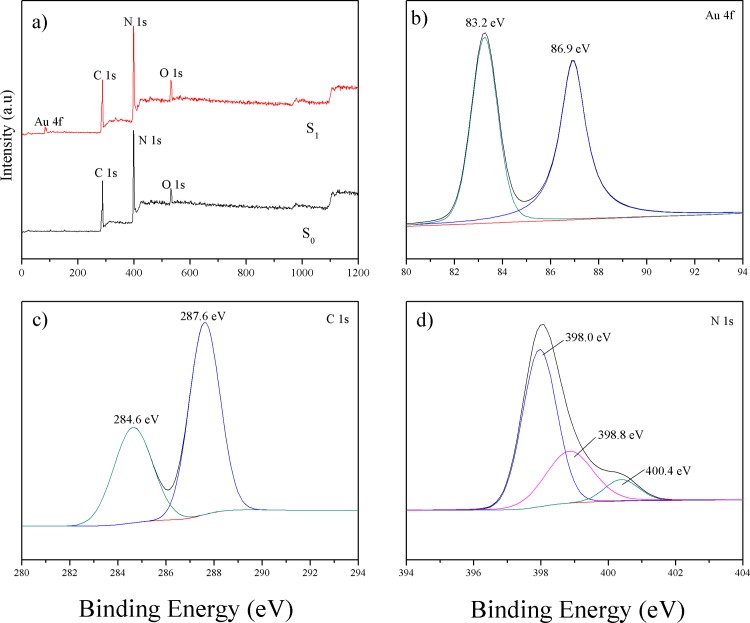
Full XPS spectra of the S_0_ and S_1_ samples (a), high-resolution spectra of the Au 4f (b), C 1s (c), and N 1s (d) for S_1_ sample.

XPS measurement is carried out to obtain the information on oxidation state and surface chemical composition of the samples. Fig 3A shows the full XPS spectra of the S_0_ and S_1_ samples. The XPS data for S_0_ is observed with sharp photoelectron peaks, confirms the presence of C and N. Additionally, a small amount of O is also observed, which may be due to surface absorption and oxidation. [21] For the S_1_ sample, besides the expected peaks of the C, N, and O elements, the XPS survey spectrum clearly reveals the presence of Au nanoparticles, suggesting that Au nanoparticles are successfully loaded on the surface of g-C_3_N_4_. No peaks for other elements are found, indicating that the S_1_ sample is primarily composed of gold, carbon, and nitrogen elements. This is consistent with the results of XRD and TEM. The two peaks centered at 83.2 and 86.9 eV in Fig 3B correspond to Au 4f_7/2_ and 4f_5/2_, [31, 32] respectively, suggesting that the Au nanoparticles exist in their metallic state. According to the XPS handbook and previous reports, [31] the binding energy values of 4f_7/2_ and 4f_5/2_ for metallic Au are centered at 84.0 and 87.7 eV, respectively. The shift of the Au 4f peaks of the S_1_ sample toward lower binding energies indicates strong interactions between Au particles and the g-C_3_N_4_ substrate. [31] Fig 3C displays the high-resolution XPS spectra of C1s. Two peaks can be distinguished at 284.6 and 287.6 eV. The major peak at 284.6 eV is exclusively assigned to C–C or adventitious carbon. [33, 34] The peak at 287.6 eV is as assigned to the C–(N)_3_ groups of g-C_3_N_4_. [35] The N1s high-resolution spectrum in Fig 3D shows an asymmetrical feature, indicating the coexistence of a number of distinguishable nitrogen environments; fitting with the three observed binding energies of 398.0, 398.8, and 400.4 eV, respectively. The three peaks are attributed to C–N = C, N–(C)_3_, and N–H. [36–38]

### Specific surface area analysis

**Fig 4:**
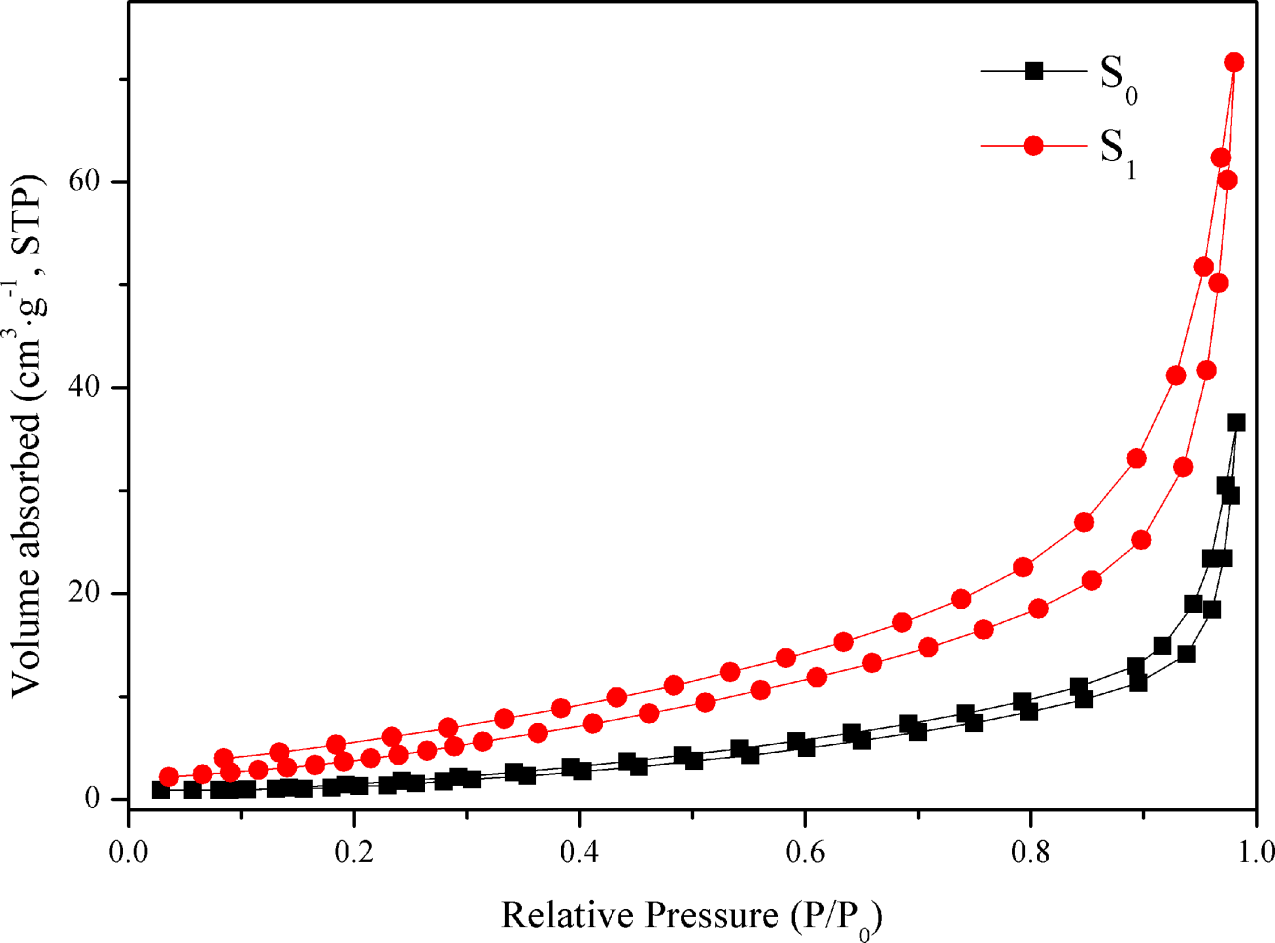
N_2_ adsorption-desorption isotherms of S_0_ and S_1_ samples.

The microstructural characteristics of the photocatalysts are conducted via full nitrogen absorption/desorption isotherms of the S_0_ and S_1_ samples, as shown in Fig 4. The curves of the products reveal that the two samples possess type III absorption/desorption isotherms, [39] which is caused by the weak adsorbent-adsorbent interaction and the existence of the nanostructures in the sample. Table 1 records the corresponding textural properties. The Brunauer-Emmett-Teller (BET) specific surface areas of the S_0_ and S_1_ samples are calculated to be 6.29 and 19.11 m^2^ g^-1^, respectively, suggesting that an optimal amount of Au nanoparticles could significantly increase the surface area of the final product. Too much Au nanoparticles aggregate on the surface of g-C_3_N_4_ and decrease the surface area of the photocatalysts. The different Au content in the samples could be responsible for the slightly different surface areas. Therefore, moderate content of Au nanoparticles plays an important role in the control over morphology of the samples.

**Table 1:** Surface properties of different samples.

Samples	*S*_bet_ (m^2^ · g^-1^)	*d*_average_ (nm)	*V*_total_ (cm^3^ · g^-1^)	Au content (wt %)
S_0_	6.29	36.04	0.06	0.00
S_5_	9.72	27.37	0.07	4.74
S_0.2_	10.75	28.92	0.08	0.18
S_2_	12.22	34.81	0.10	1.86
S_0.5_	16.96	24.08	0.10	0.47
S_1_	19.11	23.19	0.11	0.93

### UV–vis diffuse reflection spectroscopy

**Fig 5:**
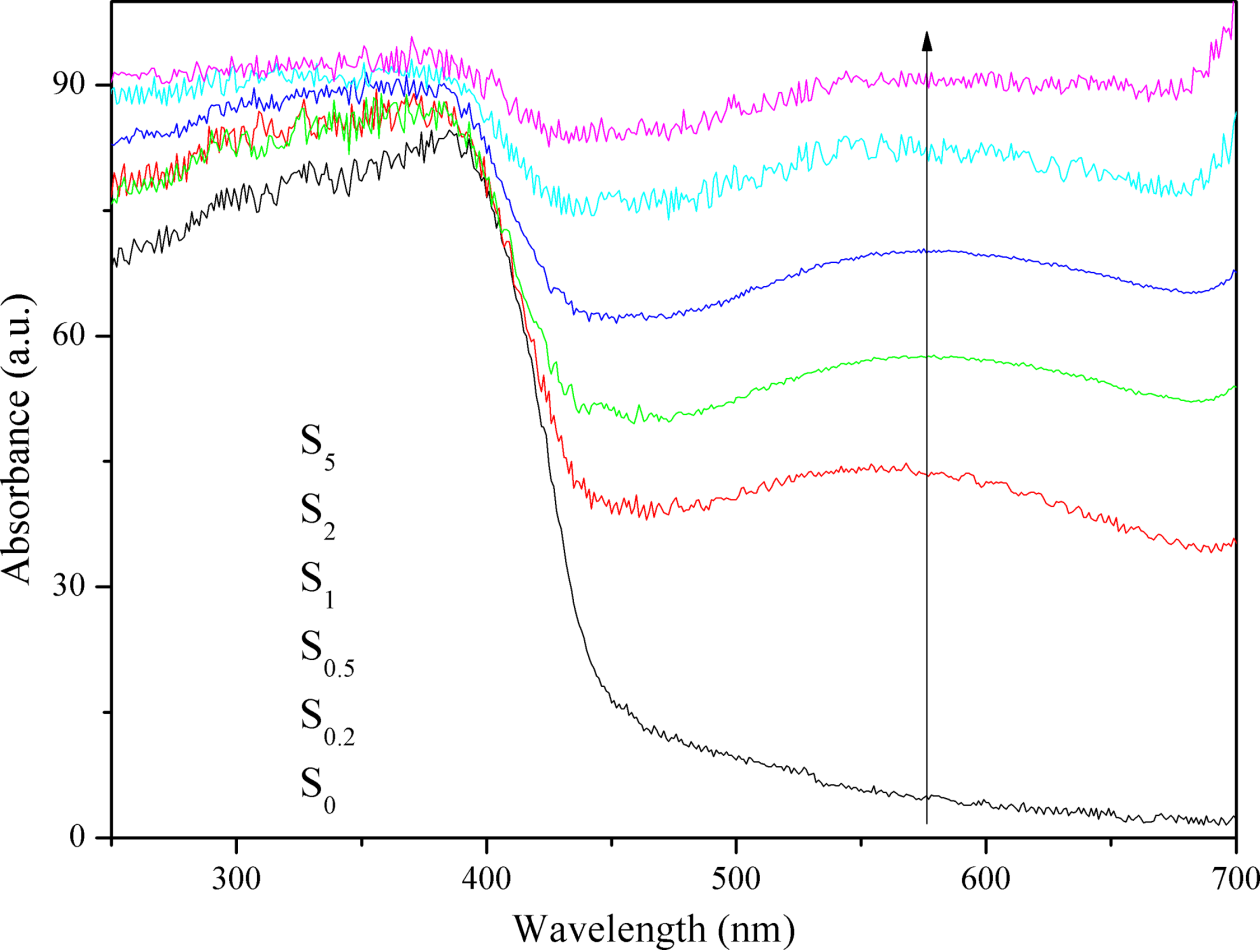
UV–vis DRS of S_0_, S_0.2_, S_0.5_, S_1_, S_2_, and S_5_.

Optical property is an important factor affecting the photocatalytic activity of the catalyst. Fig 5 exhibits the UV–vis DRS of S_0_, S_0.2_, S_0.5_, S_1_, S_2_, and S_5_. It is clear that the S_0.2_, S_0.5_, S_1_, S_2_, and S_5_ samples exhibit excellent response to visible light at ~640 nm, which is beneficial for visible light-induced production of hydrogen. According to the spectrum of S_0_, the maximum absorption wavelength is approximately 460 nm, which indicates that g-C_3_N_4_ is able to respond to visible light. The bandgap was calculated using the following equation: [40]

$$E_{g}=\frac{1240}{\lambda_{g}}$$ (1)

where *E*_g_ is the band gap energy (eV), *λ*_g_ is the crossing point between the extrapolated line tangent to the shoulder of the absorption band and the x-axis (nm). According to the formula, the bandgap of g-C_3_N_4_ is 2.7 eV and is in keeping with the previously reported value. [41] Additionally, the S_0.2_, S_0.5_, S_1_, S_2_, and S_5_ samples display a much broader absorption band compared to the pristine g-C_3_N_4_, which extends throughout the whole visible light region of 400–640 nm. A new absorption peak appears at approximately 550 nm in the visible light region, and the absorbance of S_0.2_, S_0.5_, S_1_, S_2_, and S_5_ increases gradually as the Au-loading level is increased from 0.2% to 5.0%. The new, broad peaks in the spectra of S_0.2_, S_0.5_, S_1_, S_2_, and S_5_ are attributed to the characteristic SPR absorption peak of metallic Au nanoparticles. The absorbance of single Au nanoparticles is displayed in Fig A in S1 File, which presents a high absorbance of near 100%. We use the optical absorbance of Au/SiO_2_ to instead of that of Au nanoparticles, because SiO_2_ would not absorb light, which is displayed in Fig B in S1 File. The SPR peak of Au is sensitive to the size, shape, and dispersity of the Au nanoparticles, of which the latter is closely associated with the loading amount of Au nanoparticles. [22]

### Photoluminescence

**Fig 6:**
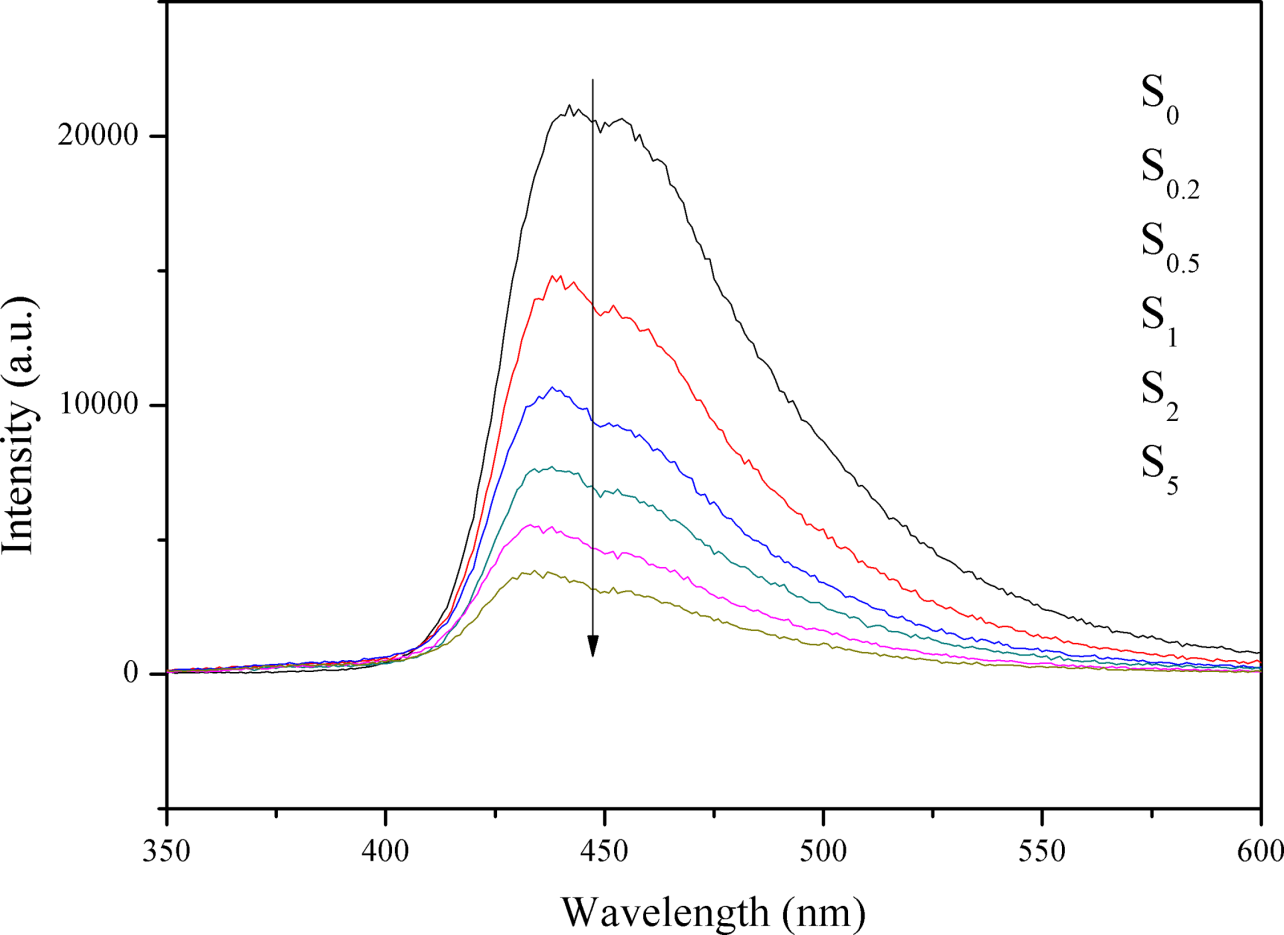
PL spectra of the S_0_, S_0.2_, S_0.5_, S_1_, S_2_, and S_5_ samples.

PL is an effective and commonly used method to investigate electron transfer in semiconductors. [42] The PL spectra of the S_0_, S_0.2_, S_0.5_, S_1_, S_2_, and S_5_ samples in the wavelength range of 350–600 nm, with the excitation light at 325 nm are shown in Fig 6. It can be seen that all the samples have similar spectral shapes and they all show intense absorption in the visible light range. The main peak at 460 nm is attributed to the emission corresponding to the band gap transition of g-C_3_N_4_. When Au is loaded on the surface of g-C_3_N_4_, the PL intensity is decreased. In general, the lower the PL intensity, the lower the recombination rate of photo-induced electron–hole pairs and the higher the photocatalytic activity of semiconductor photocatalysts. A higher loading content of Au results in the PL intensity being quenched to a higher degree. When the amount of loaded Au is greater than 1 wt %, the intensity of PL continues to decrease. The small decrease may result from the excessive loading of Au nanoparticles decreasing the fluorescence rather than from the suppressed charge recombination, which is confirmed by the reduced H_2_ evolution rates of S_2_ and S_5_. Au can form electron capture hydrazine on the surface of the catalyst as an effective cocatalyst, which prompted the photoinduced charge migrate to cocatalyst and prevented the recombination of electrons and holes.

### Amperometric i-t curves

**Fig 7:**
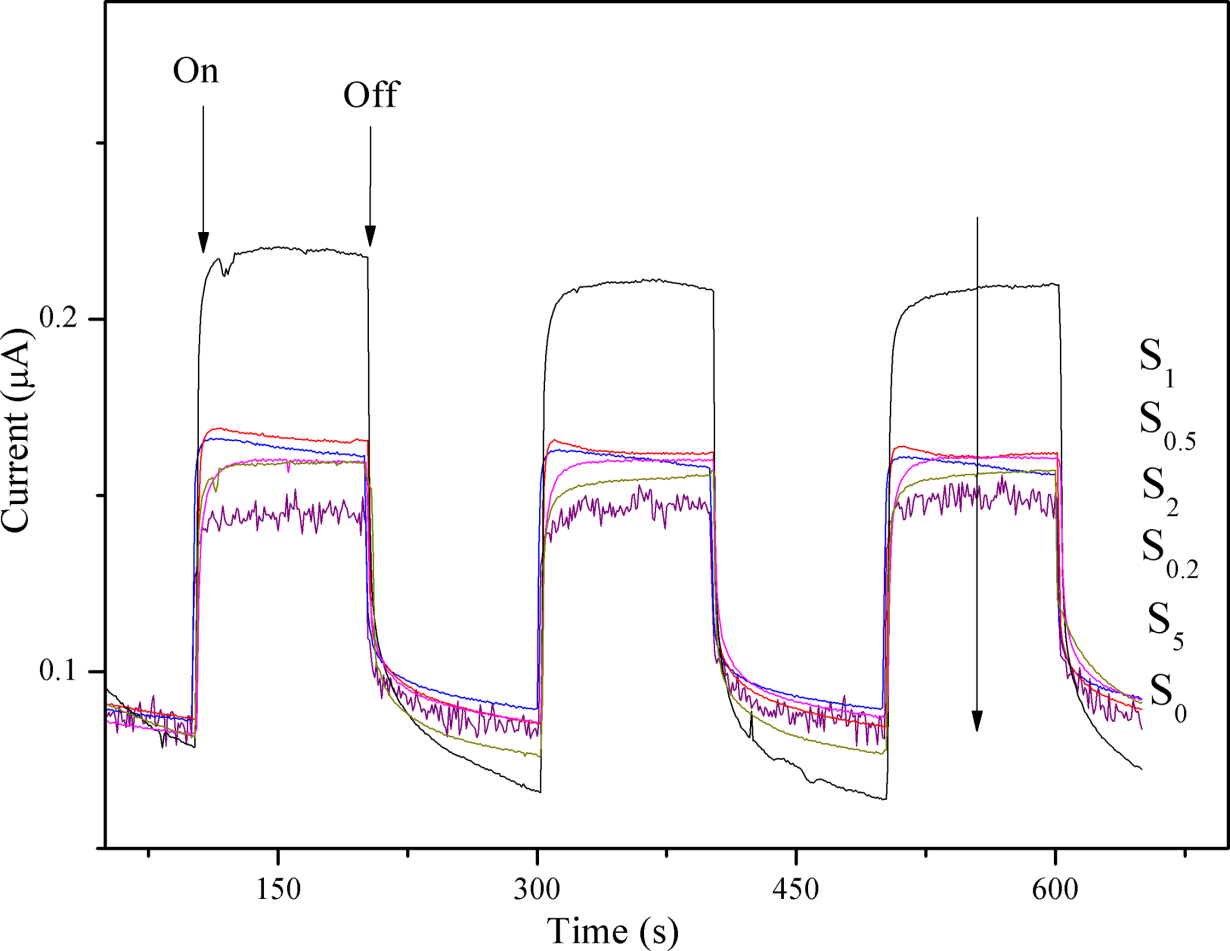
Photocurrent density of S_0_, S_0.2_, S_0.5_, S_1_, S_2_, and S_5_.

To investigate the photoinduced behavior of the generated photocurrent response of samples, the amperometric i-t curves obtained in the dark and under visible light irradiation, are shown in Fig 7. In the dark, all the samples show a negligible current response. In contrast, upon illumination, the photocurrent response is sharp increased and a steady-state current is obtained after settling. The prompt increase in photocurrent response from light-off to light-on state is mainly ascribed to the fast separation and transportation of the photogenerated electrons on the surface of the working electrodes. The photocurrent returns to background value after turning off light. All of the samples show reproducible photocurrent generation in response to illumination, and there is no obvious current drop after 600 s of testing. All tested samples with Au loaded on the surface of g-C_3_N_4_ present higher photocurrent responses with respect to the S_0_ sample, and the photocurrent density of the samples decreases in the order: S_1_ > S_0.5_ > S_2_ > S_0.2_ > S_5_ > S_0_. It can be seen that the photocurrent of the S_0.2_, S_0.5_, S_1_, S_2_, and S_5_ samples increases gradually as the content of Au is increased from 0.2 to 1.0 wt%, after which it begins to decline with further increase in the content of Au. The lower photocurrent of the S_2_ and S_5_ samples with respect to S_1_ indicates that the increased aggregation of Au nanoparticles can induce new recombination centers, thereby inhibiting further generation of electrons and holes. [29] The current generated by the S_1_ sample is ca. 0.22 μA, whereas that of g-C_3_N_4_ is ca. 0.13 μA; the photocurrent density of the S_1_ sample is nearly twice as high as that of S_0_. The loading of Au nanoparticles is beneficial for the improvement of photocatalytic activity. This is explained by two reasons: (i) the formation of a Schottky barrier facilitating electron transfer from g-C_3_N_4_ to Au, which accelerates the separation of photo-generated charge carriers, and (ii) the SPR effect induced by plasmonic Au nanoparticles, which enhances the absorption of visible light.

### Electrochemical impedance spectroscopy

**Fig 8:**
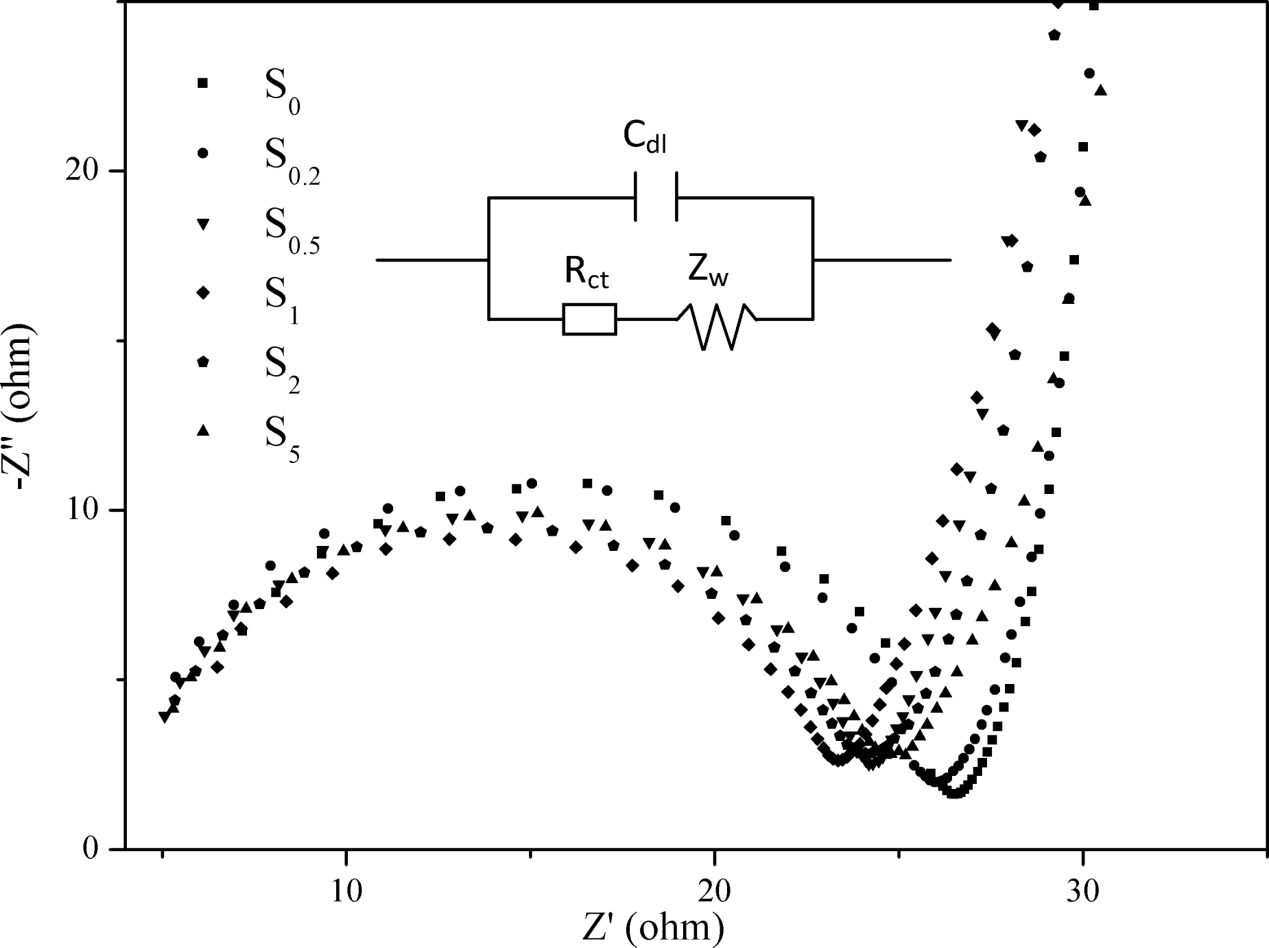
Nyquist plots of all the samples tested in the dark.

EIS is a powerful tool for characterizing charge transfer across interfaces. [43, 44] To gain insight into the interfacial resistance of the samples, EIS measurements were carried out at open-circuit voltages and in the dark. The Nyquist plots of all the samples are shown in Fig 8. A semicircle is observed at the high-to-medium frequencies, and a straight, sloping line is observed at low frequencies, as seen in the graph. The semicircle represents charge-transfer resistance, whereas the straight sloping line is associated with diffusion resistance through the bulk of the active material. [45] Obviously, the charge-transfer resistance of the S_0.2_, S_0.5_, S_1_, S_2_, and S_5_ samples is smaller than that of S_0_, which may be attributed to the higher electronic conductivity caused by the loading of Au nanoparticles. Based on the EIS data, an equivalent circuit (inset in Fig 8) can be fitted by the Zsimp Win 3.20d program with good accuracy. The equivalent circuit is used to analyze the measured impedance data. As shown in the circuitry, *R*_ct_ and *C*_dl_ represent the charge transfer resistance and double layer capacitance, respectively; and *Z*_w_ stands for the Warburg impedance associated with the diffusion process. The fitting values from this equivalent circuit are presented in Table 2. The *R*_ct_ of all the samples decreases in the order: S_1_ < S_0.5_ < S_2_ < S_5_ < S_0.2_ < S_0_. The S_1_ sample exhibits the smallest *R*_ct_ value of 18.45 Ω · cm^-2^, much lower than that of g-C_3_N_4_, which means that charge-transfer resistance is significantly reduced by Au-loading. The *C*_dl_ values display the opposite tendency as that of *R*_ct_. The low *R*_ct_ and high *C*_dl_ values of the S_1_ sample indicate high electron transfer efficiency and photocatalytic activity, which is in accordance with i-t curves.

**Table 2:** Model parameters of the photocatalysts based on EIS results.

Sample	*R*_ct_ (Ω · cm^-2^)	*C*_dl_ × 10^8^ (F · cm^-2^)	*Z*_w_ × 10^4^ (S^0.5^ · Ω^-1^ · cm^-2^)
S_0_	21.88 ± 1.13	1.43 ± 0.17	6.69 ± 0.67
S_0.2_	21.60 ± 0.98	2.02 ± 0.18	8.34 ± 0.81
S_5_	20.06 ± 0.89	2.10 ± 0.18	8.29 ± 0.75
S_2_	19.38 ± 0.91	2.11 ± 0.20	7.81 ± 0.71
S_0.5_	19.35 ± 1.01	2.30 ± 0.22	7.61 ± 0.74
S_1_	18.45 ± 1.00	2.59 ± 0.31	5.75 ± 0.49

### Incident photon-to-current conversion efficiency

**Fig 9:**
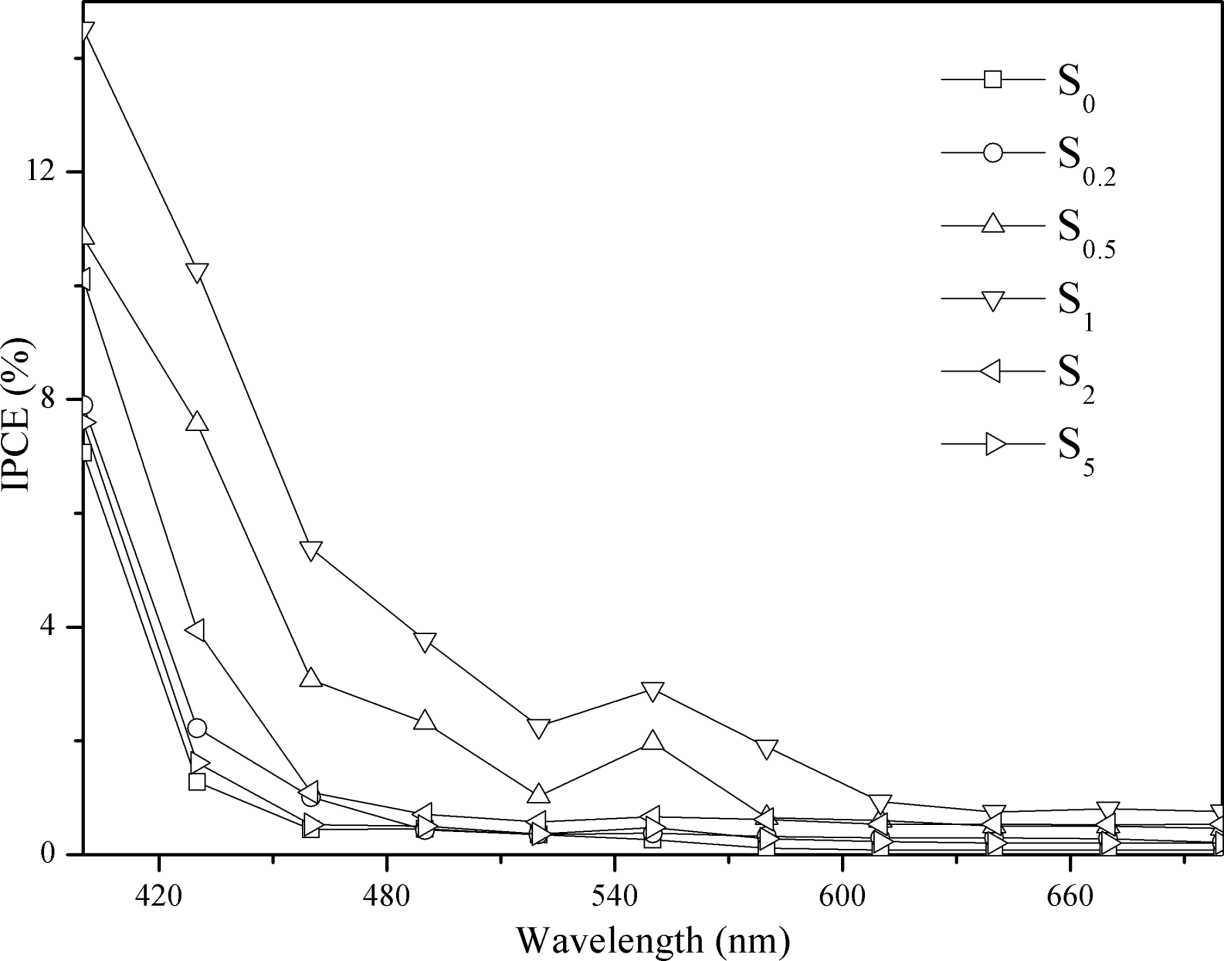
IPCE plots for S_0_, S_0.2_, S_0.5_, S_1_, S_2_, and S_5_ at 1.2 eV vs Ag/AgCl in 0.1 M Na_2_SO_4_.

To quantify the photoresponse of S_0_, S_0.2_, S_0.5_, S_1_, S_2_, and S_5_, IPCE measurements at 1.2 eV vs Ag/AgCl as the reference electrode are presented in Fig 9. IPCE can be expressed as follows: [46, 47]

$$\mathrm{IPCE}(\%)=\frac{1241\times I_{p}\times 100}{\lambda \times \varphi }$$ (2)

where *λ*, *φ*, and *I*_p_ denote the wavelength of the incident light (nm), the illumination power (mW cm^-2^), and the photocurrent density (mA cm^-2^) measured at the corresponding wavelength, respectively.

All samples show a visible light IPCE value, and the IPCE is higher at shorter wavelengths. The absorption threshold of g-C_3_N_4_ is approximately 460 nm, with an IPCE value of almost zero, which varies correspondingly with the UV–vis DRS. Introduction of Au nanoparticles into the g-C_3_N_4_ results in substantial enhancement of the IPCE in the wavelength range from 460 to 640 nm. The S_1_ sample displays significantly high IPCE values of 14.52%, 2.9%, and 0.74% under monochromatic light irradiation of 400, 550, and 640 nm, respectively. The IPCE plots increase in the order: S_0_ < S_5_ < S_0.2_ < S_2.0_ < S_0.5_ < S_1_, which is consistent with the observed i-t curves and photocatalytic activity. The IPCE values gradually increase when the content of Au nanoparticles is increased from 0.2% to 1.0%, and then decline as the content of Au nanoparticles is further increased from 1.0% to 5.0%. This result indicates that the loaded Au nanoparticles initially improve the photoelectrical performance of the g-C_3_N_4_ sample, while an excess of Au nanoparticles results in formation of agglomerates on the surface of g-C_3_N_4_; these may act as recombination centers and thereby reduce the efficiency of charge separation. In addition, the small hump in the region of 540–570 nm is caused primarily by the SPR effect of Au nanoparticles, whose absorption peak is at ∼550 nm when deposited on the surface of semiconductor photocatalysts. [48]

### Electron spin resonance

**Fig 10:**
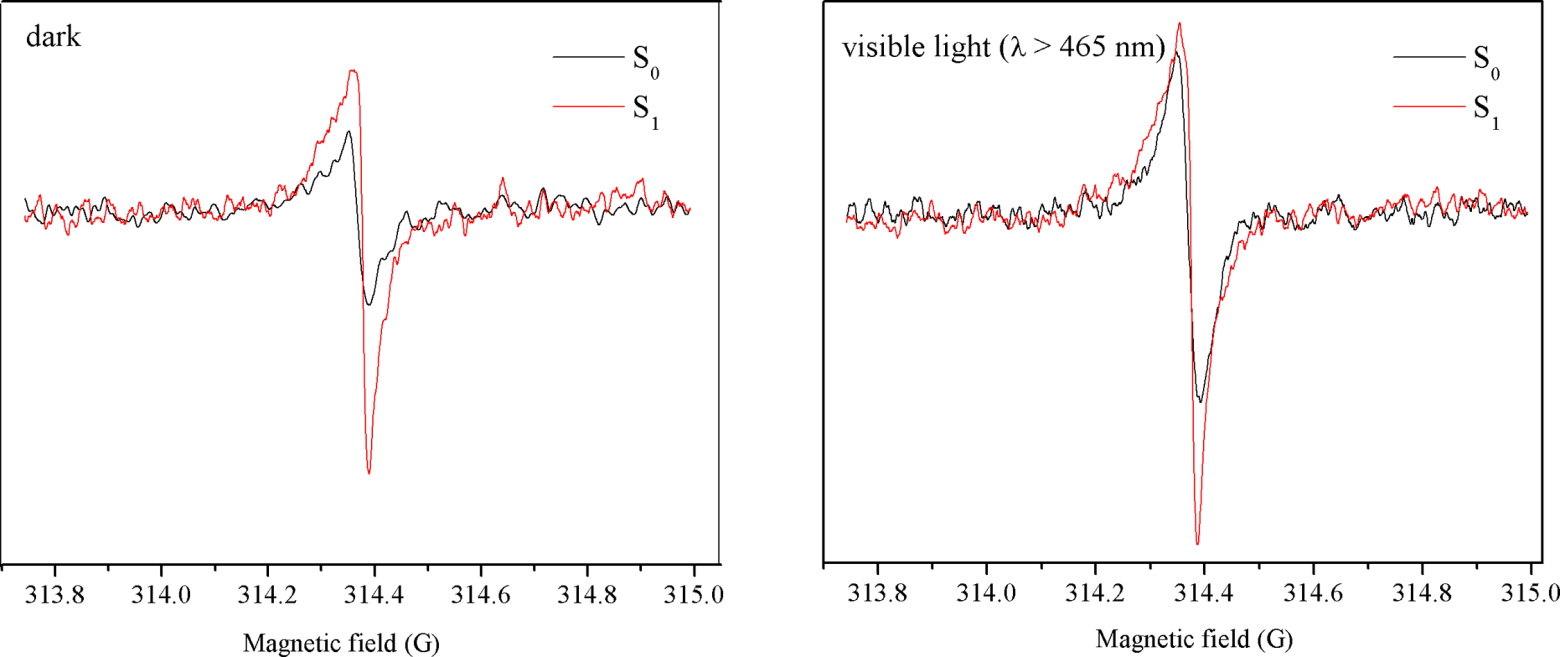
ESR spectra of the S_0_ and S_1_ samples in the dark and under visible light (*λ* > 465 nm).

ESR is a sensitive technique used to investigate the charge separation efficiency and the generation of photoactive, trapped CB electrons in samples, to further determine the influence of Au-loading on the photogenerated carriers. The ESR experiments on S_0_ and S_1_ were carried out in dark or under irradiation with visible light (*λ* > 465 nm) for 5 min at a temperature of 90 K. As depicted in Fig 10, a single Lorentzian line centered at a g-value of 2.0034 is observed for S_0_, in the dark as well as under irradiation, establishing the semiconductor structure of the sample. The Lorentzian line, according to the literature, originates from the conduction electrons generated in the localized π states of g-C_3_N_4_. [49, 50] The existence of these conduction electrons is beneficial for photocatalytic reactions. In the dark, the loading of Au nanoparticles results in the rise of the signal from trapped CB electrons. This suggests that charge separation is much more efficient in this material. In addition, S_1_ also shows a higher intensity of the CB electron signal under irradiation (*λ* > 465 nm). It must be noted that the g-C_3_N_4_ support is not capable of absorbing light in this wavelength range. Thus, the trapped electrons indicated by the higher-intensity signal may not from the CB of g-C_3_N_4_, but from the Au nanoparticles. The Au nanoparticles are able to absorb visible light, consistent with the results of UV–vis DRS.

### Photocatalytic activity

**Fig 11:**
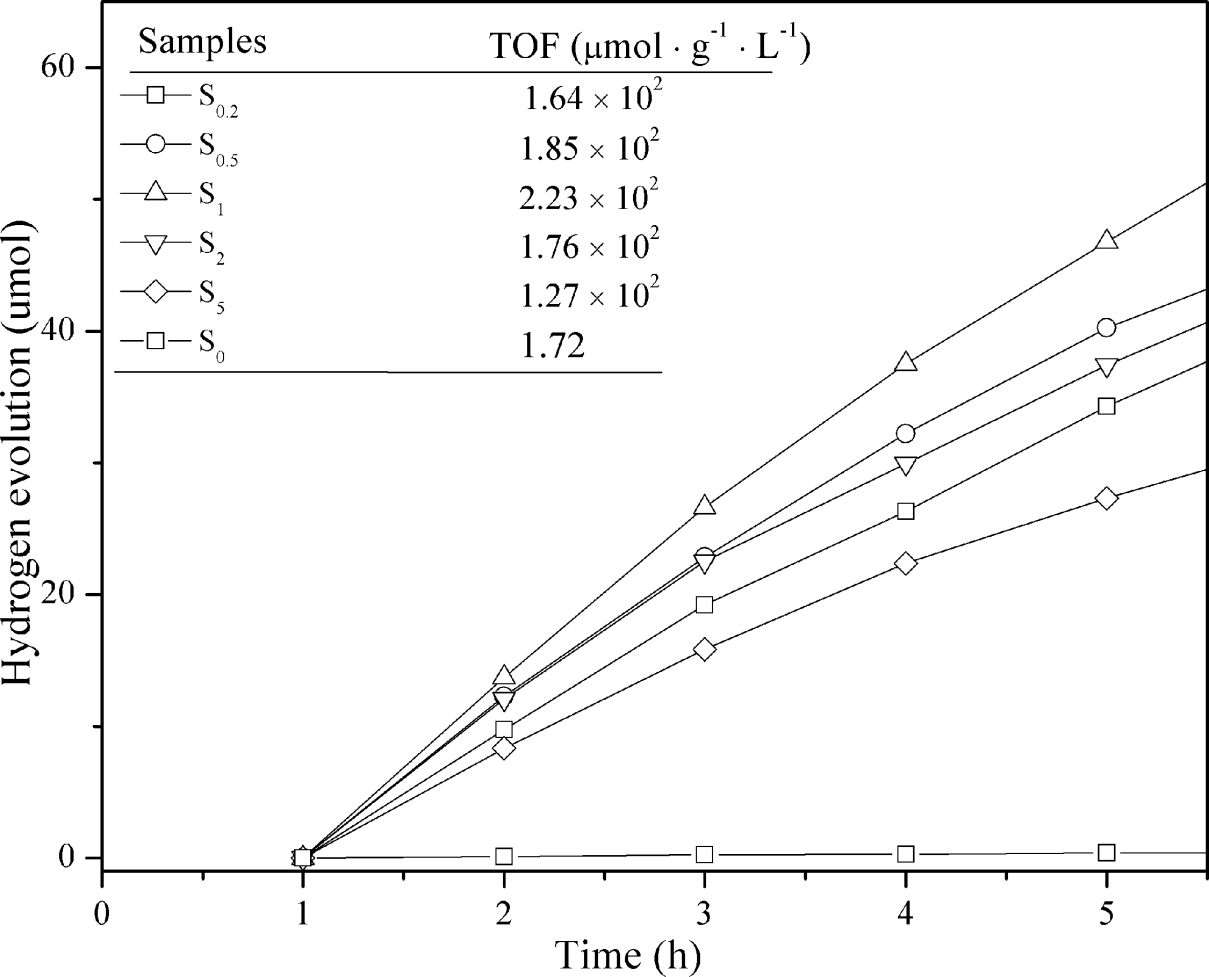
Time courses of photocatalytic hydrogen evolution under visible light (*λ* > 400 nm) on all samples.

The S_0_, S_0.2_, S_0.5_, S_1_, S_2_, and S_5_ samples exhibit stable photocatalytic activity for the production of hydrogen from water under visible light irradiation (*λ* > 400 nm). Hydrogen evolution as a function of time, during a 5 h testing period is shown in Fig 11. The measurements are carried out in a quartz container filled with methanol/water (3:7 v/v) solution for the evolution of hydrogen (methanol is used as the hole scavenger). The S_0_ sample shows low hydrogen production of approximately 0.43 μmol continuously over 5 h. This is probably due to the rapid recombination of electron−hole pairs generated through direct excitation of g-C_3_N_4_. Moreover, there is short of applicable active sites on the surface of g-C_3_N_4._ When Au is loaded on the surface of g-C_3_N_4_, a higher photocatalytic activity for hydrogen production is expected. The S_1_ sample displays an optimized TOF of 223 μmol h^-1^ g^-1^, which is nearly a 130-fold improvement over g-C_3_N_4_. The Au can facilitate charge separation at the Au/g-C_3_N_4_ interface and promote the transfer of photoexcited electrons from the g-C_3_N_4_ CB to the Au nanoparticles, inhibiting the electron–hole pair recombination process. A noticeable decrease in the hydrogen production rate is observed when an excess of Au nanoparticles is loaded (S_2_ and S_5_). This is mainly because aggregates of Au nanoparticles can induce the formation of new recombination centers; additionally, an excess of Au nanoparticles will decrease the reaction interface involved in the hydrogen production, which is in accordance with the BET results.

### Electron transfer mechanism

**Fig 12:**
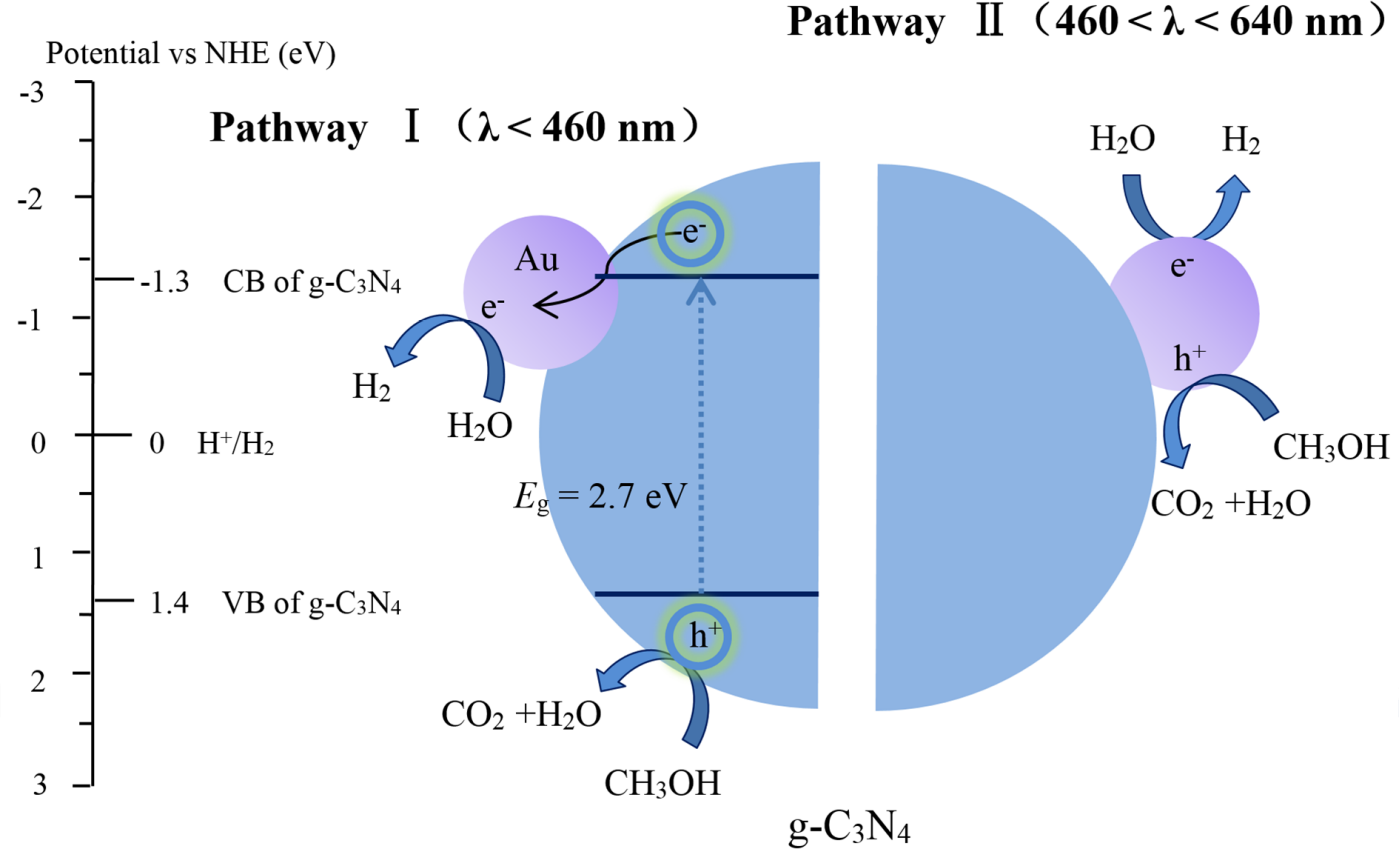
Photocatalytic mechanism of Au/g-C_3_N_4_-catalyzed hydrogen production under visible light (*λ* > 400 nm).

The rate of photocatalytic hydrogen production is approximately 130 times improved by 1 wt% loading of Au nanoparticles on the surface of pure g-C_3_N_4_. The following mechanism of photocatalytic hydrogen evolution from the reduction of water over Au/g-C_3_N_4_ under visible-light irradiation is proposed, and the schematic diagram is presented in Fig 12. There are two different mechanisms of electron transfer in Au/g-C_3_N_4_ for the reduction of water, depending on the wavelength of the incident light.

Under irradiation by the visible light spectrum (400 < *λ* < 460 nm), g-C_3_N_4_ is excited, and CB electrons and valence band (VB) holes are generated. These electrons are quickly transferred to Au, due to its lower Fermi level (0.94 V vs NHE), which results in the formation of a Schottky barrier at the interface between Au and g-C_3_N_4_. The Schottky barrier efficiently facilitates the separation of charge carriers. Holes left at the VB of g-C_3_N_4_ are quenched by the sacrificial reactant, CH_3_OH. Water is reduced to hydrogen by the electrons at the Au surface.

Au nanoparticles can harvest visible light (460 < *λ* < 640 nm) due to the SPR effect, and thus extend the range of visible light absorption from 460 to 640 nm, which is confirmed by UV-vis DRS and IPCE. Au nanoparticles absorb the resonant photons to generate “hot electrons” [51, 52] and enrich the surface of g-C_3_N_4_. The “hot electrons”, together with electrons on the CB of g-C_3_N_4_, reduce water to hydrogen.

## Conclusions

In summary, enhanced hydrogen production was achieved using the photocatalyst Au/g-C_3_N_4_, which is responsive to an extended range of wavelengths in the visible light region. Au/g-C_3_N_4_ is fabricated by a facile, photo-assisted reduction method. The g-C_3_N_4_ sample photo-sensitized by Au nanoparticles exhibits a significantly enhanced hydrogen evolution TOF value of 223 μmol g^-1^ h^-1^, which is 130 times higher than that of g-C_3_N_4_. According to the XRD results, the Au-loading affects neither the morphology nor the crystal structure of the g-C_3_N_4_ photocatalysts. XPS results confirm that the Au species loaded on the surface of g-C_3_N_4_ is metallic Au. Au/g-C_3_N_4_ exhibits strong light absorption and an extended region of visible light absorption from 460 to 640 nm, which is confirmed by UV–vis DRS and IPCE. The high photocatalytic activity of Au/g-C_3_N_4_ is attributed to the following reasons. Firstly, the lower Femi level of Au nanoparticles leads to electron transfer from the CB of g-C_3_N_4_ to the Au nanoparticles, which suppresses the recombination of electron–hole pairs. In addition, higher visible light absorption due to the SPR effect in gold nanoparticles results in the production of a large number of photogenerated electrons, which is confirmed by ESR. The Au/g-C_3_N_4_ composite is a promising photocatalyst which provides a new potential material for photocatalytic applications.

## Supporting Information

S1 File Methods and Figures. **Fig A in S1 file.** The UV–vis DRS of S_0_, S_0.2_, S_0.5_, S_1_, S_2_, S_5_, and Au nanoparticles. (In order to measure the optical property of Au nanoparticles, we loaded Au nanoparticles on the surface of SiO_2_. And the mass fraction of Au is about 5%. The absorbance of SiO_2_ is nearly zero, so there is no effect on the absorbance measurement of Au nanoparticles.) **Fig B in S1 File.** The UV–vis DRS of SiO_2_ and Au/SiO_2_. (It confirms that the optical absorbance of SiO_2_ is nearly zero.)
(DOC)
