# Supplementary material for: Enhanced Hydrogen Evolution in the Presence of Plasmonic Au-Photo-Sensitized g-C3N4 with an Extended Absorption Spectrum from 460 to 640 nm
Source: PLoS One. 2016 Aug 30;11(8):e0161397. doi: 10.1371/journal.pone.0161397 (PMC5004922; doi:10.1371/journal.pone.0161397)
Supplement: S1 File — Fig A in S1 file. The UV–vis DRS of S0, S0.2, S0.5, S1, S2, S5, and Au nanoparticles. (In order to measure the optical property of Au nanoparticles, we loaded Au nanoparticles on the surface of SiO2. And the mass fraction of Au is about 5%. The absorbance of SiO2 is nearly zero, so there is no effect on the absorbance measurement of Au nanoparticles.) Fig B in S1 File. The UV–vis DRS of SiO2 and Au/SiO2. (It confirms that the optical absorbance of SiO2 is nearly zero.) (DOC) [file pone.0161397.s001.doc]

**Supplementary Information**

Enhanced Hydrogen Evolution in the Presence of Plasmonic Au-photo-sensitized g-C_3_N_4_ with an Extended Absorption Spectrum from 460 to 640 nm

Lihong Xie, Zhuyu Ai, Meng Zhang, Runze Sun, and Weirong Zhao**[[1]](#footnote-2)***

Department of Environmental Engineering, Zhejiang University, Hangzhou 310058, China

The supporting information consists of 2 pages, including 2 figures.

**1. Materials and methods**

1.1. Materials

Silicon dioxide (SiO_2_), chloroauric acid tetrahydrate (AuCl3·HCl·4H2O), methanol (CH3OH), were purchased from Sinopharm Chemical Reagent Co., Ltd, China. All reagents were analytical grade and used without further purification. And Deionized water was used in the experiment.

1.2 Fabrication of Au/SiO_2_ photocatalyst

In order to insure the accuracy of experiment, the preparation of Au/SiO_2_ nanoparticles which is as same as that of Au/g-C_3_N_4_ are as follows: SiO_2_ (0.30 g) was dispersed in a methanol/water (1:4 v/v) solution with a total volume of 75 mL in a double-layer photoreaction cell. A certain volume of AuCl_3_∙HCl∙4H_2_O solution (10 mg mL^-1^) was then added to the cell as the gold precursor, and magnetic stirred for 2 h to mix homogeneously. The resulting suspension was irradiated under a 300 W Xe lamp with a wavelength range of 200–400 nm for 3 h under continuous stirring. The final product was separated by filtration, washed three times with distilled water, and dried in an oven at 60 °C for 12 h.

**2. Figures**

Fig A

Fig B

1. ^^ Corresponding author. Tel.: +86-571-8898-2032; fax: +86-571-8898-2032.

   E-mail address: weirong@mail.hz.zj.cn [↑](#footnote-ref-2)
